# A needs-based methodology to project physicians and nurses to 2030: the case of the Kingdom of Saudi Arabia

**DOI:** 10.1186/s12960-021-00597-w

**Published:** 2021-04-26

**Authors:** Samantha Gailey, Tim A. Bruckner, Tracy Kuo Lin, Jenny X. Liu, Mohammed Alluhidan, Taghred Alghaith, Hussah Alghodaier, Nabiha Tashkandi, Christopher H. Herbst, Mariam M. Hamza, Nahar Alazemi

**Affiliations:** 1 School of Social Ecology, University of California Irvine, Irvine, CA USA; 2 Program in Public Health, University of California Irvine, Irvine, CA USA; 3 Department of Clinical Pharmacy, Medication Outcomes Center, University of California San Francisco, San Francisco, CA USA; 4 Department of Social and Behavioral Sciences, Institute for Health and Aging, University of California San Francisco, San Francisco, CA USA; 5 Saudi Health Council, Riyadh, Saudi Arabia; 6 Lancaster University, Lancashire, UK; 7 Ministry of National Guard-Health Affairs, Riyadh, Saudi Arabia; 8 World Bank, Washington, DC USA

**Keywords:** Workforce planning, Needs based, Epidemiologic model, Kingdom of Saudi Arabia, Human resources for health

## Abstract

**Background:**

The Kingdom of Saudi Arabia (KSA), as part of its 2030 National Transformation Program, set a goal of transforming the healthcare sector to increase access to, and improve the quality and efficiency of, health services. To assist with the workforce planning component, we projected the needed number of physicians and nurses into 2030. We developed a new needs-based methodology since previous global benchmarks of health worker concentration may not apply to the KSA.

**Methods:**

We constructed an epidemiologic “needs-based” model that takes into account the health needs of the KSA population, cost-effective treatment service delivery models, and worker productivity. This model relied heavily on up-to-date epidemiologic and workforce surveys in the KSA. We used demographic population projections to estimate the number of nurses and physicians needed to provide this core set of services into 2030. We also assessed several alternative scenarios and policy decisions related to scaling, task-shifting, and enhanced public health campaigns.

**Results:**

When projected to 2030, the baseline needs-based estimate is approximately 75,000 workers (5788 physicians and 69,399 nurses). This workforce equates to 2.05 physicians and nurses per 1000 population. Alternative models based on different scenarios and policy decisions indicate that the actual needs for physicians and nurses may range from 1.64 to 3.05 per 1000 population in 2030.

**Conclusions:**

Based on our projections, the KSA will not face a needs-based health worker shortage in 2030. However, alternative model projections raise important policy and planning issues regarding various strategies the KSA may pursue in improving quality and efficiency of the existing workforce. More broadly, where country-level data are available, our needs-based strategy can serve as a useful step-by-step workforce planning tool to complement more economic demand-based workforce projections.

**Supplementary Information:**

The online version contains supplementary material available at 10.1186/s12960-021-00597-w.

## Background

Human resources play a crucial role in delivering health services. From a policy- and country-level perspective, health planners and decision-makers must ensure that the right number of people, with the right skills, deliver appropriate health services for the population’s health needs. Given the substantial training and resources required to develop a health workforce, planners that can anticipate health workforce needs into the future will be better equipped to match these resources to the particular demographic and health characteristics of the population.

From a planning perspective, a foundational component to understanding the overall workforce that is needed requires an estimation of the health conditions of the population. Whereas several strategies have been proposed to arrive at estimates of an ideal volume and skill mix of the health workforce, most begin with trying to understand the population’s morbidities that are amenable to cost-effective health care treatments and interventions. Some countries, for instance, have undergone population ageing, which increases the median age of the population and shifts the age structure into older ages. This ageing process reflects both the current age structure of the population (i.e., baby boomers reaching older ages) and lower fertility rates. As a result, “ageing” countries show an increasing predominance of chronic diseases as the leading causes of disability and death. Other populations may have a greater prevalence of risky behaviors (e.g., smoking) that produce a unique set of associated illnesses. Regardless of a country’s specific demographic and health behavior profile, planning for an efficient health workforce should involve matching the health workforce to the distribution of health conditions in that population that health workers will address.

Starting in 2016, The Kingdom of Saudi Arabia (KSA) developed the ambitious National Transformation Program designed to achieve key objectives by 2030 which focus on 8 themes. Transformation of the healthcare sector in this high-income country serves as the first theme of the “Vision 2030” strategic framework. A core element of this transformation in the KSA involves increasing access to, and improving the quality and efficiency of, health services [1].

The fragmented nature of health subsystems in the KSA poses challenges to long-term strategic workforce planning. Although health care is currently provided free of charge to all Saudi citizens, the KSA is in the process of restructuring their healthcare system to privatize some health services for citizens and non-citizens [2]. Health services are delivered through hospitals and outpatient facilities operated by multiple public sector subsystems and, increasingly, the private sector. These facilities include approximately 500 hospitals (nearly one-third of which are operated by the private sector), 2000 public primary health centers, and 3000 private clinics and polyclinics. Specialized health centers also provide a range of health services, including dental, cardiology, and oncology [3].

Over the past decade, the health workforce in the KSA has grown steadily across all sectors. From 2011 to 2019, the total health workforce increased from 303,578 to 467,650. In 2019, nurses comprised 43% (199,013) of all staff, allied health workers (including applied medical specialists and technicians) 26% (123,619), physicians 20% (94,335), and pharmacists 7% (31,872) [4, 5].

To help the KSA achieve their goals, we projected the needed number of physicians and nurses into 2030. The use of the term “need” in HRH planning, however, remains ambiguous in the literature. In their literature review, Tomblin Murphy and colleagues [6] note that many studies using the term needs-based employ “need”, “demand”, and “utilization” interchangeably. This circumstance creates a challenge when country stakeholders and academics alike attempt to compare their estimates of HRH to that of other countries (or academics) who use fundamentally different methods and assumptions to arrive at estimates of HRH need [7, 8]. For this reason, we clarify that our needs-based projections use, as the base inputs, estimates of the true prevalence of health conditions (rather than treated prevalence or observed healthcare utilization patterns). Our projections, moreover, are grounded in epidemiology of health conditions and therefore do not consider economic factors such as cost, availability, and willingness to pay.

We developed a new needs-based methodology, rather than using earlier estimates, since previous global ratio-based benchmarks of health worker concentration may not apply to the KSA. Previous estimates of physicians and nurses (per 1000 population) that are needed include 2.28, taken from the World Health Organization (WHO) World Health Report 2006 [9], and 4.5, taken from WHO Sustainable Development Goals 2016 [10]. These benchmarks, however, may not apply to the KSA, given that they were derived from a regression-based exercise using population averages of data on 110 countries, before 2010, and mostly in low- and middle-income contexts with a high burden of infectious diseases. In addition, the 4.5 value assumes a national strategy of universal health coverage in which health care services, even for non-priority health conditions, are subsidized by the federal government. This strategy appears inconsistent with the current restructuring of the Saudi healthcare system and expansion of the private sector [2]. Neither the 4.5 nor the 2.28 estimates, moreover, are based on detailed epidemiologic evidence of the prevalence of health conditions in a particular country. For these reasons, the relevance of these benchmarks to the needs-based estimates of health workers in the KSA remains unknown.

We used an epidemiologic “needs-based” approach to estimate, to 2030, the number of physicians and nurses needed to treat the burden of disease in the KSA. This approach has been used previously by governmental and non-governmental organizations [11–14] but, in our view, remains underutilized by specific countries. The benefit to the planner of a needs-based approach involves the important goal-setting exercise that answers the following question: what is the expected number of physicians and nurses needed to deliver cost-effective interventions and treatments to adequately address the health needs of the population?

Whereas we focus our forecasting exercise on the KSA, the needs-based methodology can be applied more broadly to other countries intending to efficiently match health workers with the health needs of the changing population. In addition, our needs-based approach innovates this field in 3 ways. First, we forecast the need into the future (i.e., 10-year horizon) using assumptions about the demography of the population. Second, we devise treatment service modules for chronic diseases that impose a large burden of disability and death in and outside of the KSA. Third, we develop a disability-adjusted life year (DALY) multiplier that allows us to scale estimates of health workers to the entire country’s burden of disease, rather than to a smaller set of conditions.

## Methods

**Fig. 1:**
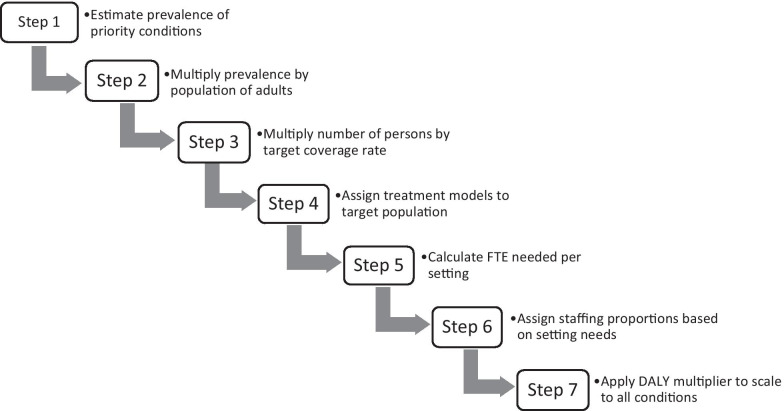
Methodological steps to forecast the number of physicians and nurses needed in the KSA to 2030. *DALY* disability-adjusted life year, *FTE* full-time equivalent

We proceeded through the following 7 steps to arrive at forecasts of physicians and nurses needed to treat the health conditions of the KSA population to 2030 (see Fig. 1). These steps, adapted from previous literature [14], resulted in 3 different scenarios, each of which considers a range of policy decisions.

(1) We retrieved data on the prevalence of priority health conditions in the KSA, including ischemic heart disease, cerebrovascular disease (stroke), major depressive disorder, diabetes mellitus, chronic obstructive pulmonary disease (COPD), and congenital anomalies, from national health surveys, peer-reviewed literature, and the Global Burden of Disease (GBD) study. (2) We multiplied prevalence estimates by the total population of adults in the KSA to derive the number of cases per condition per year. (3) We assigned treatment coverage goals to each of the 6 priority conditions to estimate the target population that may seek care. (4) We applied service delivery models of cost-effective interventions, based on results of WHO-CHOICE (Choosing Interventions that are Cost-Effective) analyses and cost-effectiveness studies in comparable countries, to estimate outpatient and inpatient visits. (5) We converted the number of visits into full-time equivalent (FTE) heath workers needed to treat priority conditions across settings (i.e., inpatient and outpatient) and (6) applied staffing ratios to each treatment setting to obtain quantities of physicians and nurses. (7) We scaled up health workers needed to treat all health conditions in the KSA (i.e., moving beyond the 6 studied priority conditions).

To account for critical uncertainty related to the burden of disease contributed by priority conditions, we applied several DALY multipliers in scale-up models, resulting in 3 different scenarios. We describe each of these steps, and key policy decisions that may influence estimates, in further detail below.

### Prevalence of priority conditions

A needs-based approach uses the prevalence of priority health conditions as its foundation to drive estimates of the size of the health workforce [11, 14–16]. We therefore used, as the starting point, information on priority health conditions contained in the KSA’s Health Sector Transformation Strategy. This strategy document, delivered by the Ministry of Health as part of “Vision 2030” for the KSA, states that, “Particular areas of concern include heart disease, stroke, diabetes mellitus, respiratory disease, mental health, road traffic accidents and congenital diseases…” (p. 9) [17]. Because we expect that preventive measures initiated by 2030 are envisaged to dramatically reduce the number of road traffic accidents, road traffic accidents were excluded for the purposes of this analysis and scenario calculations.

Health conditions within these “areas of concern” were further specified, as the service delivery models required to treat these conditions vary widely. Health conditions were selected according to the following criteria: (a) they impose substantial disability, morbidity, or mortality in the KSA; (b) epidemiologic data and estimates of the condition are available; and (c) the condition is amenable to cost-effective treatment delivered in a primary care or hospital setting. This process resulted in a list of 6 priority health conditions: ischemic heart disease, stroke, major depressive disorder, diabetes mellitus, COPD, and congenital anomalies (including congenital heart defects, neural tube defects, cleft lip and palate).

The best available estimates of prevalence for these 6 priority health conditions, by age group, were used to derive the population that may seek care (Table 1)  (Fig. 1, Step 1). Prevalence estimates from national health surveys in the KSA, including the General Authority for Statistics (GASTAT) Household Health Survey [18] and the Saudi Health Interview Survey (SHIS) [19] were prioritized. The GASTAT Household Health Survey, conducted in 2018, is a nationally representative survey targeting all individuals (Saudis and non-Saudis) who reside in the KSA. The survey sample was chosen by identifying 24,012 households that represent the survey population. The SHIS, conducted in 2013, is based on a multistage representative sample of 10,735 adults ages 15 years and older. To ensure estimates are as context relevant as possible, recent data sources were preferred to older ones. For example, for diabetes prevalence, the GASTAT Household Health Survey was preferred to the SHIS as the latter is 5 years older.

**Table 1:** Population-based estimates of prevalence for priority health conditions and their sources

Priority health condition	Prevalence (%)	Source
Ischemic heart disease	5.50	Al-Nozha et al. [21]
Cerebrovascular disease	0.65	GBD study 2017 [22]
Major depressive disorder	2.78	GBD study 2017 [22]
Diabetes mellitus	8.50	GASTAT 2018 [18]
COPD	2.40	Wali et al. [20]
Congenital anomalies	1.24	GBD study 2017 [22]

*COPD* chronic obstructive pulmonary disease, *GASTAT* General Authority for Statistics, *GBD* Global Burden of Disease

Other prevalence estimates including those for COPD and ischemic heart disease rely on Saudi cross-sectional, population-based surveys described in peer-reviewed literature. For COPD, data were collected between June 2010 and December 2011 using a random digit dialing survey (*n* = 10,001) [20]. A community-based survey, conducted between 1995 and 2000 (*n* = 17,232), was used to estimate the prevalence of ischemic heart disease [21]. In certain cases, where national estimates were not available, the GBD study [22] database was used. Prevalence estimates for the latest year for all ages and both sexes were used.

### Total cases

Prevalence estimates for priority health conditions were then multiplied by the United Nations population projections for years 2020–2030 [23] to derive possible cases per condition per year (Fig. 1, Step 2). This calculation assumes no change in prevalence of these conditions to 2030.

### Treatment coverage targets

Information on plausible treatment coverage rates was then applied to each of the priority health conditions (Fig. 1, Step 3). Target coverage rates were determined on the basis of the severity of the health condition, the ability of a clinician to detect cases, and the probability that cases will seek care. Based on these factors, and consistent with the approach used in the literature in devising these targets [24–26], the following treatment coverage rates were established (and applied across all years): 80% for ischemic heart disease, stroke, COPD, diabetes mellitus, and congenital anomalies; and 33% for major depressive disorder. A relatively high treatment coverage target was assigned to cardiovascular diseases, COPD, diabetes mellitus, and congenital anomalies due to the large burden associated with these conditions in the KSA and, for acute events, the severity of symptoms and ease of detection. Conversely, a relatively low target coverage rate was assigned to major depressive disorder given that patients may not report symptoms and clinicians in a primary care setting may fail to detect cases [27]. We also assessed the potential impact of a health education campaign that would increase treatment coverage levels (see "Scenarios and policy decisions" below).

### Service delivery models

Next, for each condition, we applied service delivery models of cost-effective interventions to estimate the number of outpatient and inpatient visits per year (Table 2) (Fig. 1, Step 4). These models were based on results of WHO-CHOICE [28] regional analyses and cost-effectiveness studies in upper-middle- and high-income countries, which best reflect the economic circumstance of the KSA [14, 24, 26]. Treatment models were determined based on (a) the percentage of cases needing care in each service setting (i.e., inpatient and outpatient), (b) the average annual number of visits per person, and (c) whether or not visits require a bed (see Additional file 1: Table S2). The WHO-CHOICE [28] cost-effective modules assume that most cases receive treatment at a primary care level, and that patients with more severe, complex, or acute conditions receive treatment at a hospital level.

**Table 2:** Needs-based estimates of outpatient visits and inpatient bed-days for priority health conditions in the KSA, 2030

Health condition	Target population	Total annual outpatient visits	Total annual inpatient bed-days
Cardiovascular diseases	1,525,397	3,050,794	3,050,794
Major depressive disorder	284,431	1,498,950	207,634
Diabetes	2,108,272	4,216,544	843,309
COPD	595,277	1,506,050	357,166
Congenital anomalies	25,544	6,386	109,839
Total	4,538,921	10,278,724	4,568,742

*COPD* chronic obstructive pulmonary disease

### Health workforce staffing

Using these outpatient and inpatient visit estimates, treatments were converted into the FTE number of physicians and nurses needed to treat priority health conditions (Fig. 1, Step 5). Workforce requirements for outpatient services were calculated using WHO estimates of workforce capacity [14, 28]. Models assumed that physicians and nurses work, on average, 225 days per year and have 11 consultations per day. The total number of expected outpatient visits was divided by 2475 (225 × 11) to obtain an estimate of the number of FTE physicians and nurses needed for outpatient care. To derive the number of FTE physicians and nurses required to meet inpatient needs, bed-days were estimated assuming that hospitals operate at 85% capacity [14]. This correction factor (1.15) was applied to obtain the target number of inpatient bed-days. Models assumed no change in FTE inputs across years.

### Staffing ratios

Staffing ratios were then applied to each treatment setting. Models assumed that physicians and nurses perform 1.7% and 98.3% of tasks in outpatient settings, and 10% and 90% of tasks in inpatient settings, respectively (Fig. 1, Step 6) [14, 24, 28]. We also assessed how task-shifting (i.e., where tasks are shifted from physicians to nurses) would affect needs-based health worker estimates (see "Scenarios and policy decisions" below).

### Scale-up models

Health workers were then scaled up to treat *all* health conditions by applying a DALY multiplier to the subset of priority health conditions studied (Fig. 1, Step 7). Scale-up models assumed that, on average, the health workforce required to treat a health condition is proportional to its contribution to the total burden of disease, as measured by DALYs, in the KSA.

This assumption was derived from data on the use of health services, retrieved from the FutureDocs Forecasting Tool [29], and the GBD study [22] for select health conditions in the US and KSA. The FutureDocs Forecasting Tool estimates the capacity of the health workforce to meet the use of health services in the US from 2011 to 2030 [29]. We compared the proportion of inpatient and outpatient visits, as a percentage of all visits [29], to the proportion of the burden of disease, as a percentage of the total burden of disease [22], for several priority conditions. For example, respiratory diseases (e.g., COPD) cause (a) 95,683,118 inpatient and outpatient visits (6.83% of total visits), and (b) 6,465,807 DALYs (6.43% of total DALYs); and mental disorders (e.g., major depression) cause (a) 75,235,774 inpatient and outpatient visits (5.37% of total visits) and (b) 5,371,622 DALYs (5.34% of total DALYs) in the US per year. Additional examples demonstrating the proportionality assumption underlying the use of a DALY multiplier to scale up health workers appear in the Additional file 1: Table S3.

Using information available in peer-reviewed literature [30] and results of the GBD study [22], the 6 studied priority health conditions in the KSA were estimated to account for 20% of the total burden of disease in the KSA. Based on the proportionality assumption, the number of FTE staff needed to treat priority conditions was multiplied by a factor of 5 to obtain the total number of FTE health workers required to treat all health conditions.

### Alternative scenarios and policy decisions

We projected, from 2020 to 2030, needs-based health worker estimates under various assumptions about scaling, resulting in 3 scenarios. Within each scenario, we further assessed the potential role of policy decisions related to task-shifting and treatment coverage rates on the estimated number of physicians and nurses needed to treat the epidemiologic needs of the population. These scenarios and policy decisions are intended to provide planners and policymakers with a range of health workforce planning options—of which a subset may be feasible given political and economic constraints.

### Scenarios

The DALY multiplier of 5 (Scenario A) assumes that the burden of disease contributed by the 6 priority conditions accounts for 20% of the total burden of disease in the KSA [22, 30]. We applied 2 additional DALY multipliers (4 and 6) to account for critical uncertainty related to this assumption. This process resulted in 3 different scenarios, where multiplication by a factor of 5 produces a median, or baseline, estimate of required health workers (Scenario A), and 4 (Scenario B) and 6 (Scenario C) provide a plausible range of health workers based on under- or overestimates of the burden of disease contributed by priority conditions.

### Policy decisions

*Staffing ratios* Next, alternative staffing ratios, which assume that a percentage of tasks (30% or 50%) can be “shifted” from physicians to nurses, were applied. Staffing ratios based on task-shifting decisions were adjusted relative to the baseline staffing ratios described above. For instance, if 30% of tasks were shifted from physicians to nurses, then physicians would perform approximately 1.2% of tasks in outpatient settings and 7% of tasks in inpatient settings.

*Treatment coverage rates* Lastly, a speculative policy option was considered, in which a health education campaign would increase absolute treatment coverage rates for all health conditions by 10%. The assumption of this policy is that persons with these conditions would be more likely to seek care. The resulting target coverage rates include 90% for ischemic heart disease, stroke, COPD, diabetes mellitus, and congenital anomalies, and 43% for major depressive disorder.

**Fig. 2:**
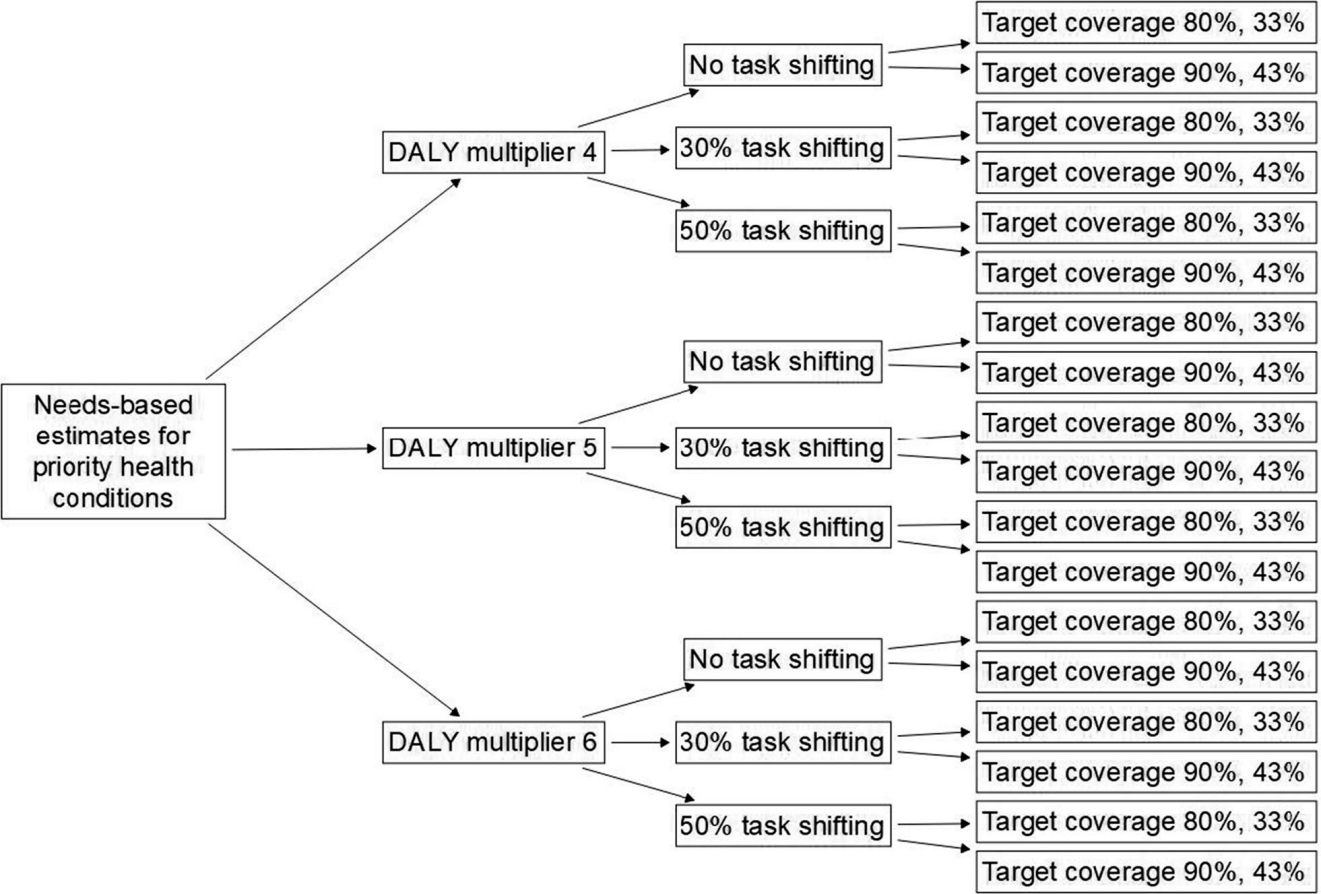
Schematic diagram of 18 needs-based estimates under various assumptions of scaling, task-shifting, and target coverage level. *DALY* disability-adjusted life years

Modeling all possible combinations of assumptions and corresponding inputs resulted in 3 scenarios (3 DALY multipliers), and 6 policy decisions (3 staffing ratios × 2 levels of target coverage) per scenario, totaling 18 workforce estimates (see schematic diagram in Fig. 2).

## Results

### Baseline model

Our baseline needs-based estimates (Model 1) are based on Scenario A (DALY multiplier of 5) and do not account for policy decisions that may increase task-shifting and/or treatment coverage levels. Specifically, the baseline model assumes that (a) priority health conditions account for 20% of the total burden of disease in the KSA, (b) no task-shifting occurs, and (c) 80% of expected cases receive treatment for cardiovascular diseases, diabetes, COPD, and congenital anomalies, and 33% for major depressive disorder.

**Fig. 3:**
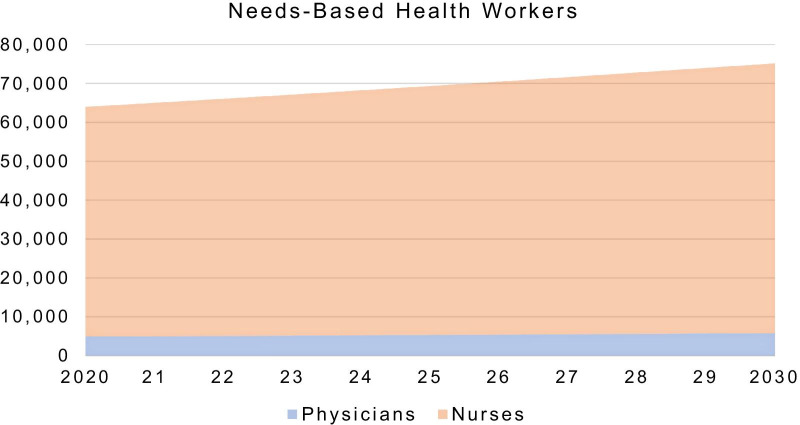
Annual needs-based physician and nurse estimates (based on Model 1) in KSA, 2020–2030

Under these assumptions, approximately 64,000 FTE nurses and physicians are needed in 2020 to meet epidemiological needs. Using the needs-based approach, it is estimated that, of these FTE workers, 4935 physicians and 59,054 nurses are required. These values equate to 2.01 health workers (physicians and nurses) per 1000 population. When projected to 2030, this FTE estimate rises to approximately 75,000 workers (5788 physicians and 69,399 nurses). This workforce equates to 2.05 physicians and nurses per 1000 population in 2030. Figure 3 provides an overview of needs for each year, between 2020 and 2030, based on Model 1.

### Alternative scenarios and policy decisions

**Fig. 4:**
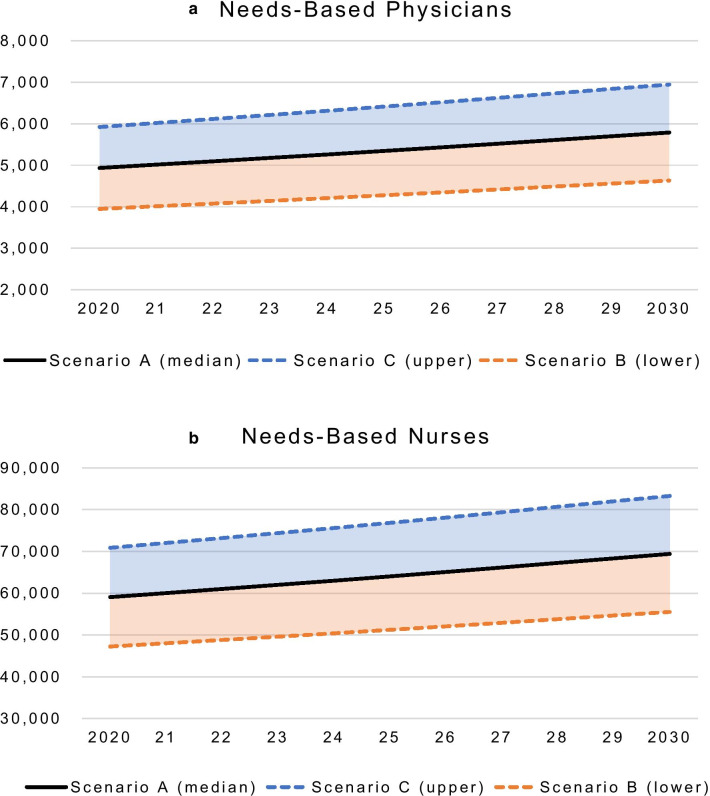
Annual needs-based **a** physician and **b** nurse estimates and scenario-based ranges* in the KSA, 2020–2030. *Scenario estimates do not include policy decisions; Scenario A = Model 1, Scenario B = Model 7, Scenario C = Model 13

Models that take into account different scenarios and policy decisions reveal that the baseline needs for FTE physicians and nurses could be close to 75,000, with a plausible range of between 60,000 and 112,000, in 2030 (see Table 3). Changes in the DALY multiplier reflect changes to the percentage of the total burden of disease accounted for by priority health conditions. Figure 4 shows the range of estimates produced under different assumptions about scaling, where Scenarios B and C produce upper- and lower-limits of estimated needs-based health workers, respectively.

**Table 3:** Needs-based estimates of the number of FTE physicians and nurses in 2030 according to model assumptions

Scenario			Policy decisions		Needs-based health workers			
DALY multiplier		Model	Task-shifting	Target coverage	Physicians	Nurses	Total	Per 1000 pop
A Median	5	1*	–	–	5788	69,399	75,187	2.05
		2	–	+ 10%	6989	86,412	93,401	2.54
		3	30%	–	4052	71,135	75,187	2.05
		4	30%	+ 10%	4892	88,508	93,401	2.54
		5	50%	–	2894	72,293	75,187	2.05
		6	50%	+ 10%	3494	89,906	93,401	2.54
B Lower limit	4	7	–	**–**	4631	55,519	60,150	1.64
		8	–	+ 10%	5591	69,129	74,720	2.03
		9	30%	–	3241	56,908	60,150	1.64
		10	30%	+ 10%	3914	70,807	74,720	2.03
		11	50%	–	2315	57,835	60,150	1.64
		12	50%	+ 10%	2796	71,925	74,720	2.03
C Upper limit	6	13	–	**–**	6946	83,279	90,225	2.46
		14	–	+ 10%	8387	103,694	112,081	3.05
		15	30%	–	4862	85,363	90,225	2.46
		16	30%	+ 10%	5871	106,210	112,081	3.05
		17	50%	–	3473	86,752	90,225	2.46
		18	50%	+ 10%	4193	107,887	112,081	3.05

*DALY* disability-adjusted life years, *pop* population

*Results of primary baseline model

Some models within each scenario consider the role of a policy decision that would increase task-shifting—that is, enhancements in certifications or in training among nurses may increase task-shifting to between 30 and 50%, reducing the ratio of physicians to nurses. For example, under Scenario A, enacting a 30% task-shifting policy (Model 3) produces needs-based estimates of 4052 physicians and 71,135 nurses. Some models also account for the potential impact of an enhanced public health campaign. Model 2, which assumes a 10% increase in treatment coverage under Scenario A, for example, produces needs-based estimates of 6989 physicians and 86,412 nurses.

## Discussion

Regression-based estimates of health worker densities that are required to treat the epidemiologic needs of a population are limited in that they typically ignore the specific country’s demographic age structure, prevalence of health conditions, and workforce planning goals. Here we detail a needs-based methodology to calculate, and forecast, the number of physicians and nurses needed to treat a country’s health conditions. We focused on the KSA because this country aims to achieve transformational goals for its health workforce by 2030. These methods, however, can be widely applied to other countries interested in estimating workforce needs based on the prevalence of health conditions in their population.

The needs-based modeling in the KSA shows that the needs for FTE physicians and nurses in 2030 range between 60,000 and 112,000 depending on the scenario and policies enacted; however, we expect the actual need is closer to 75,000 health workers. This range translates into physician and nurse densities from 1.64 to 3.05 per 1000 population. These densities fall within the general range of earlier ratio-based benchmarks reported by WHO in the World Health Report 2006 (i.e., 2.28) and the Universal Health Coverage Report of 2016 (i.e., 4.5) [9, 10]. Importantly, the current modeling exercise provides a level of granularity to the KSA and uses detailed epidemiologic estimates for one country. For this reason, the current needs-based exercise for the KSA represents a substantial improvement relative to the use of population-level regression averages that rely on low- and middle-income country contexts and assume a social goal of universal health coverage for all.

The estimated number of FTE health workers needed is below the total stock of physicians and nurses in the KSA, but above the stock of Saudi national physicians and registered nurses. According to recent data [4, 5], when counting both Saudi and non-Saudi nationals, as well as registered/bachelor and enrolled/diploma nurses, there are approximately 300,000 physicians and nurses in the KSA, which equates to about 9 health care workers per 1000 population. This estimated health workforce exceeds the needs-based estimates provided above (though the needs-based approach does not account for economic factors that may also drive the supply of workers). At the same time, the needs-based estimate does not exceed current stock if accounting only for Saudi nationals and separating registered nurses from diploma nurses (i.e., those who do not have a college degree in nursing). Only about 100,000 of the physicians and nurses are Saudi, which equates to about 3 physicians and nurses per 1000 population. Without diploma nurses, the density of Saudi physicians and nurses is only 1.4 per 1000 population.

Whereas the KSA may not face an overall shortage of physicians and nurses in 2030, the literature suggests that a shortfall of general practitioners remains possible [4, 5]. Approximately 22% of the KSA’s physician workforce classify as generalists (defined as general practitioners or family medicine physicians working in primary care) and 74% as specialists. By contrast, in OECD countries, on average, 30% and 63% of physicians work as generalists and specialists, respectively [4, 5].

Additionally, the KSA may face a mismatch among specialists, where some specialties appear overrepresented relative to demand. For example, the KSA has above average levels of surgical specialists, obstetricians/gynecologists (OBGYNs), and pediatricians, but below average levels of psychiatrists and endocrinologists. Post-graduate training, moreover, is skewed toward specialties with current overcapacity. To address the potential shortage of primary care doctors and achieve its strategic objectives, the KSA may need to increase its share of generalists (e.g., from 22% to nearer the OECD average of 30%) and encourage growth in underrepresented specialties [4, 5].

Limitations of needs-based estimates include that, while they are useful for workforce planning, they rely on several assumptions. A key assumption is that the “input” data used (e.g., on prevalence of conditions, treatment coverage, worker productivity) are accurate. Additionally, models are initially based on prevalence estimates of 6 priority health conditions that account for approximately 20% of the disease burden and workforce effort in the KSA [22, 30]. This approach may yield different results than one which attempts to enumerate the disease burden for each and every condition that affects the Saudi population, or an approach that uses as its basis a set of conditions that require a greater share of workforce effort. Moreover, we applied a range of DALY multipliers to account for inherent uncertainty in scale-up models. However, future work may benefit from assessing the validity of alternative DALY multipliers based on the capacity of the health workforce to meet the health needs of the population (e.g., a less efficient workforce may require more physicians and nurses to treat the same disease burden).

We also note a scarcity of evidence on cost-effective staffing ratios, particularly in allied health professions [31]. For this reason, and to simulate the influence of policies that encourage task-shifting, we applied several staffing ratios. However, the most cost-efficient and effective ratio of physicians to nurses likely varies by healthcare setting, country, and condition. Future research that examines workforce ratios, particularly in the growing areas of mental health services and diabetes care, would be useful for planning health workforce requirements into the future.

In addition, we cannot compare treatment coverage goals set for each priority condition to current levels of coverage in the KSA. Although information on condition-specific treatment coverage is limited, the KSA does provide recent estimates of the total percentage of the population covered by the healthcare system, by age group and nationality (Saudi/non-Saudi) [18]. According to these data, in 2018 38.6% of the total population of the KSA (i.e., across age group and nationality) had health insurance coverage [18]. Our condition-specific treatment coverage goals exceed overall healthcare coverage in the KSA, but treatment coverage likely varies substantially by condition. Planners would benefit from having information on treatment coverage by health condition, rather than in the aggregate, so that policymakers could set reasonable goals for gradually improving treatment coverage for priority conditions.

All of the scenarios modeled in the KSA assume no worsening in the burden of chronic disease by 2030, other than due to population ageing, which may be optimistic. Several projections about the burden of chronic disease in the KSA into 2030 and beyond portend an increase in diabetes, stroke, and heart disease, among other conditions [32, 33]. These increases may arise in part from the ageing of the population and continued increases in obesity-related chronic diseases. To the extent that these trends continue, the overall prevalence of heart disease, stroke, and diabetes could increase over time, and our workforce needs-based estimates might therefore be underestimated, regardless of the other assumptions made. Careful attention and monitoring are warranted to understand these conditions into the future. If these conditions continue to increase in prevalence as expected, then policy efforts that focus specifically on cardiovascular diseases and diabetes may be necessary.

Additionally, when we developed our needs-based methodology, the coronavirus disease (COVID-19) did not contribute substantially to the current or anticipated disease burden in the KSA. Accounting for the COVID-19 pandemic may change the number of physicians and nurses needed to treat the unique epidemiologic needs of the population in 2020–2021. However, communicable diseases, like traffic accidents (as discussed above in "Background"), appear more amenable to reduction through population-level prevention strategies and public health efforts than through health care treatments. We expect that, with improved population-level prevention and widespread vaccination, the pandemic will not affect the 2030 forecast.

Planners should also be reminded that estimates do not account for patient preferences. In the KSA context, patients may prefer a particular gender, Saudi national vs. foreign health provider, nurse vs. physician, or resident vs. consultant. In this exercise, we assumed that health care worker productivity can be applied equally to all patients regardless of preferences. This assumption may be politically and socially untenable and/or undesirable for the Saudi population from a “demand-side” perspective.

The important question of ideal worker “mix” remains an issue in the KSA and elsewhere. There remain unresolved questions about the benefit/cost calculations of employing and training physicians vs. nurses, the optimal level of task-shifting, the quality of training programs for various worker specialties, and (in some countries) goals to reduce the reliance on foreign workers. All of these issues represent important policy and economic considerations. Our needs-based estimates, therefore, should be placed within the larger context of the mix of public and private investments, as well as patient preferences, regarding what gender, worker type, and skill mix would be ideal for a particular country.

Wealthy neighboring countries with similar demographics to the KSA may benefit from this analysis. The Gulf Cooperation Council (GCC) countries share common challenges associated with health care workers’ availability and distribution [34–36]. Furthermore, evidence shows very similar patterns of non-communicable diseases and associated risk factors in the GCC countries [37–40]. This approach may therefore be utilized as a viable tool for anticipating the right number of physicians and nurses needed into the future in any other member country of the GCC.

## Conclusion

Needs-based forecasts provide a visioning exercise in that they attempt to answer the question: how many workers would it take to achieve a desired level of healthcare coverage for all members of society? Our baseline model shows that approximately 75,000 workers (5788 physicians and 69,399 nurses)—2.05 workers per 1000 population—will be needed to meet the epidemiologic needs of the Saudi population in 2030. Based on this projection, the KSA will not face a needs-based health worker shortage in 2030. However, alternative estimates based on scenarios assuming different levels of population health, and policy decisions that take into account changes in task-shifting and enhanced public health campaigns, suggest that the actual needs for FTE physicians and nurses could range from 60,000 (1.64 per 1000 population) to 112,000 (3.05 per 1000 population).

Estimates of needs-based workforce densities depend strongly on how need is defined [6, 41]. Previous HRH literature, for instance, has used need to describe not only the health workforce required to treat the population burden of disease, but also patient demand, willingness to pay, and health care supply. Our approach and our definition of “needs-based” applies a population lens that focuses on the overall disease burden and may promote agendas. However, this approach contrasts economic, or “willingness to pay,” approaches which consider the economic aspects of price, supply, and demand [6, 7]. Our approach also assumes equitable and efficient allocation of health services to all, which in some countries may not be realistic.

To this end, our needs-based approach should be viewed as epidemiological, rather than economic. Where country-level data are available, we encourage the use of needs-based forecasts as a complement to labor market demand-based forecasts. Furthermore, integration of preventive and public health efforts as part of the Vision 2030 Strategy, which focuses on the social and economic determinants of health, may crucially affect workforce need. Taken together, these multi-sector strategies will assist with setting appropriate workforce goals and planning accordingly for 2030.

## Supplementary Information

**Additional file 1: Table S1.** All population-based estimates of prevalence for priority health conditions (color-coded by data source). **Table S2.** Service delivery model exemplars for priority conditions. **Table S3.** Use of health services and burden of disease for selected health conditions in the United States.

## Data Availability

Not applicable.

## Abbreviations

COPD: Chronic obstructive pulmonary disease
DALYs: Disability-adjusted life years
FTE: Full-time equivalent
GASTAT: General Authority for Statistics
GBD: Global Burden of Disease
GCC: Gulf Cooperation Council
GHDx: Global Health Data Exchange
KSA: Kingdom of Saudi Arabia
SHIS: Saudi Health Interview Survey
WHO: World Health Organization
