# Supplementary material for: A needs-based methodology to project physicians and nurses to 2030: the case of the Kingdom of Saudi Arabia
Source: Hum Resour Health. 2021 Apr 26;19:55. doi: 10.1186/s12960-021-00597-w (PMC8072319; doi:10.1186/s12960-021-00597-w)
Supplement: Supplementary file 1 — Additional file 1: Table S1. All population-based estimates of prevalence for priority health conditions (color-coded by data source). Table S2. Service delivery model exemplars for priority conditions. Table S3. Use of health services and burden of disease for selected health conditions in the United States. [file 12960_2021_597_MOESM1_ESM.docx]

**Supplementary Material**

**Appendix Table S1.** All population-based estimates of prevalence for priority health conditions (color-coded by data source).

| **Ischemic heart disease** | **Cerebrovascular disease (stroke)** | **Major depressive disorder** | **Diabetes mellitus** | **Chronic obstructive pulmonary disease** | **Congenital anomalies** |
| --- | --- | --- | --- | --- | --- |
| 2.35 | **0.65*** | **2.78*** | 5.46 | 1.80 | **1.24*** |
| **5.50*^1^** |  | 3.0 (males);  9.0 (females)** | 13.40 | **2.40*^2^** |  |
|  |  |  | **8.50*** |  |  |
|  |  |  | 17.72 |  |  |

***** Selected prevalence estimate

** Lifetime prevalence

| **Source:** |
| --- |
| Global Burden of Disease (GBD) Study, 2017 |
| Primary Source Literature |
| Saudi Health Interview Survey (SHIS), 2013 |
| General Authority for Statistics (GASTAT) Household Health Survey, 2018 |
| World Bank Health Nutrition and Population Statistics, 2017 |
| Saudi National Mental Health Survey, 2019 |

**Primary Source Literature**

1. Al-Nozha MM, Arafah MR, Al-Mazrou YY, Al-Maatouq MA, Khan NB, Khalil MZ, Al-Khadra AH, Al-Marzouki K, Abdullah MA, Al-Harthi SS, Al-Shahid MS. Coronary artery disease in Saudi Arabia. Saudi med J. 2004 Sep 1;25(9):1165-71.
2. Wali SO, Idrees MM, Alamoudi OS, Aboulfarag AM, Salem AD, Aljohaney AA, Soliman MH, Abdelaziz MM. Prevalence of chronic obstructive pulmonary disease in Saudi Arabia. Saudi Med J. 2014 Jul 1;35(7):684-90.

**Appendix Table S2.** Service delivery model exemplars for priority conditions.

| **Priority condition** | **Source** | **Location in source text** |
| --- | --- | --- |
| Heart disease / stroke (combined) | Salomon et al. (2012) [Technical Appendix] | Main text (pp. 19-20) |
| Heart disease (acute) | Berger et al. (2008) | Figure 1. Hospital length of stay stratified by sample year (p. 7) |
| Stroke (acute) | Kwok et al. (2012) | Table 1. Characteristics of study population [length of stay] (p. 728) |
| Major depressive disorder | Bruckner et al. (2011) |  |
| Diabetes mellitus | Salomon et al. (2012) [Technical Appendix] | Table A25. Annual quantities of inpatient bed-days and outpatient visits for diabetes (p. 25) |
| Chronic obstructive pulmonary disease (COPD) | Salomon et al. (2012) [Technical Appendix] | Table A18. Annual quantities of inpatient bed-days and outpatient visits for COPD (p. 17) |
| Congenital anomalies | Higashi et al. (2015) | Table 2. Burden of congenital anomalies amenable to surgery in low- and middle-income regions (p. 234) |
| Cleft lip | Lee, Yen, & Allareddy (2018) | Table 2. Length of stay and total charges – all ages and by age groups (p. 532) |
| Congenital heart disease | Silberbach, Shumaker, Menashe, Cobanoglu, & Morris (1993) | Table 2. Influence of preoperative conditions on hospital charge and postoperative length of stay (p. 960) |

**Appendix Table S3.** Use of health services and burden of disease for selected health conditions in the United States.

|  | **Use of healthcare services** | | | | **Burden of disease** | |
| --- | --- | --- | --- | --- | --- | --- |
| Cause | Inpatient | Outpatient | All Setting | % total use | DALYs | % total DALYs |
| Respiratory | 3,329,320 | 92,353,798 | 95,683,118 | 6.83% | 6,465,807 | 6.43% |
| Congenital | 1,119,520 | 3,615,114 | 4,734,634 | 0.34% | 952,072 | 0.95% |
| Musculoskeletal | 2,972,067 | 130,025,772 | 132,997,839 | 9.50% | 9,682,811 | 9.63% |
| Mental | 2,853,839 | 72,381,935 | 75,235,774 | 5.37% | 5,371,622 | 5.34% |
| All cause | 37,726,020 | 1,362,399,266 | 1,400,125,286 |  | 100,563,718 |  |
